# The British Hospitals Association and the Act

**Published:** 1912-12-07

**Authors:** 


### THE BRITISH HOSPITALS ASSOCIATION AND THE ACT.

Those of our readers who are members, or connected with voluntary hospitals, will have received a circular letter dated the 20th ultimo, signed by the Chairman of the Council, Dr. Mackintosh. It indicates the cautious policy pursued by the responsible officers of this Association at the present crisis. It may prove of great importance that no time should be lost in collecting the necessary information to enable the Council of the Association to meet, with a view to decide the important points which have arisen, affecting as they do the action of the voluntary hospitals in relation to insured persons after January 15, 1913. We hope, therefore, that the authorities of each hospital will realise the importance of promptly replying to the questions accompanying the Chairman's circular letter, for this course will be of material assistance to the Council of the British Hospitals Association at the present time. The circular letter is as follows:—

"As medical benefit will come into force on January 15, 1913, and no provision has yet been made for institutional treatment of insured persons, it would greatly assist the Council of this Association if you would kindly send the Honorary Secretary your replies to the questions on the enclosed schedule. When the Council is in possession of this information, they will be in a better position to approach the Chancellor of the Exchequer, with a view to showing him the position in which the voluntary hospitals are placed.

"I would also request you to inform me whether the honorary medical staff of your hospital passed any resolution in reference to the treatment of insured persons, either as in- or out-patients, after January 15, 1913, and if so, I should be glad to receive a copy of such resolution."

With reference to the last paragraph it will be very helpful for every voluntary hospital, of whatever size, to send to the Honorary Secretary of the British Hospitals Association, 14 Belsize Park Gardens, London, N.W., a copy of any resolution in reference to the treatment of insured persons, which may have been passed by the honorary medical staff of their institution, with the date on which such resolutions were adopted, and an intimation as to how far they have been made effective in regard to (*a*) in-patients or (*b*) out-patients, or both, from January 15, 1913.

The following is the schedule enclosed in Dr. Mackintosh's letter:—

BRITISH HOSPITALS ASSOCIATION.

Name of Hospital ..........................................................

In-Patients.

Total number of In-patients under treatment ..................

from ..................................... to.....................................

Total number of Insured Persons included in the above

...........................................................................

Out-Patients.

Total number of Out-Patients treated ..........................

from ..................................... to .....................................

Total number of Insured Persons included in the above

...........................................................................

*Date* 1912. *Signed* ..............................

N.B.—Should you have already kept these records, please give the results for as long a period as possible. If you have as yet kept no such records, would you be so kind as to keep a record for *one* week, beginning as soon as practicable after the receipt of this form?

Please fill in and return this form to Mr. Conrad W. Thies, the Honorary Secretary, 14 Belsize Park Gardens, London, N.W.

Here, again, we urge every secretary or the chairman of each voluntary hospital, of whatever size, to fill in a return in the form given, setting forth the particulars asked for. The importance of this consists in the fact that the completer the list of hospitals making this return, the greater will be the value of the information which the British Hospitals Association are at present engaged in obtaining from voluntary hospitals throughout Great Britain. All forms when filled up should be placed in an envelope and directed to the Hon. Secretary at the address given above.

The Association at Work.

We are glad to learn that a meeting of the Executive Committee was held on the 30th ultimo, when the position of the voluntary hospitals in regard to insured persons was brought under notice. There is a feeling, we believe, that it is desirable to delay action until it is known what arrangements, if any, have been come to between the members of the medical profession and the Chancellor of the Exchequer. The importance of these negotiations nobody can deny; but they relate primarily and mainly to the treatment of insured persons in their own homes by members of the medical profession. They should not, except indirectly, affect the position of the voluntary hospitals towards the insured persons who may apply for free in-patient treatment on and after January 15, 1913. It is true that the Act contemplates the supply of adequate medical treatment through private practitioners to every insured person in their own homes. It is, however, fully understood by those who have studied the Act and know the facts, that there is no guarantee that insured persons, when seriously ill, can be treated adequately in their own homes. Indeed, it must be clear to everyone who has a wide knowledge of the circumstances, under which the majority of insured people live, that it will be often impossible to provide adequate medical treatment for insured persons in their own homes, in great cities especially.

Now the *pro rata* system provides for an agreement to be entered into between the managers of each voluntary hospital and the approved society, or societies, which may wish to arrange for in-patient accommodation for their members on a business basis. The actual sum to be paid by the approved societies to each voluntary hospital for the accommodation in question will represent the actual money spent upon each such case for maintenance and treatment in the hospital, plus 10 per cent. to defray the cost of medical attendance. These arrangements would leave the voluntary principle untouched, place the managers of each hospital in a position to provide adequately for the medical treatment of the societies' members as in-patients at the hospital, and so safeguard the interests of everybody concerned. It is clear from the answers given by Mr. Masterman in the House of Commons on the 27th ultimo, published in another column, that the Government recognise the *pro rata* system. It was further stated by Mr. Lloyd George, in his speech to the Hospital Deputation on July 25, 1911, that "it was absolutely essential that the voluntary hospitals should not be hurt at all (by the Insurance Act), because they were the only places where any progress was made in medicine, and the only places where poor people could be treated for a very serious illness. No Government could allow a single hospital to be closed." Let all interested in the voluntary hospitals note carefully these encouraging actions and statements whilst making preparations for the future.

A letter from the secretary explaining what steps the Association is taking will be found on page 283.

